# Nucleosome Remodeling by Fun30^SMARCAD1^ in the DNA Damage Response

**DOI:** 10.3389/fmolb.2019.00078

**Published:** 2019-09-04

**Authors:** Susanne C. S. Bantele, Boris Pfander

**Affiliations:** Max Planck Institute of Biochemistry, DNA Replication and Genome Integrity, Martinsried, Germany

**Keywords:** Fun30/SMARCAD1, nucleosome remodeling, DNA double-stranded break, DNA end resection, cell cycle, post-translational modification, genome stability

## Abstract

Many cellular pathways are dedicated to maintain the integrity of the genome. In eukaryotes, the underlying DNA transactions occur in the context of chromatin. Cells utilize chromatin and its dynamic nature to regulate those genome integrity pathways. Accordingly, chromatin becomes restructured and modified around DNA damage sites. Here, we review the current knowledge of a chromatin remodeler Fun30^SMARCAD1^, which plays a key role in genome maintenance. Fun30^SMARCAD1^ promotes DNA end resection and the repair of DNA double-stranded breaks (DSBs). Notably, however, Fun30^SMARCAD1^ plays additional roles in maintaining heterochromatin and promoting transcription. Overall, Fun30^SMARCAD1^ is involved in distinct processes and the specific roles of Fun30^SMARCAD1^ at DSBs, replication forks and sites of transcription appear discordant at first view. Nonetheless, a picture emerges in which commonalities within these context-dependent roles of Fun30^SMARCAD1^ exist, which may help to gain a more global understanding of chromatin alterations induced by Fun30^SMARCAD1^.

Fun30 (function unknown now, budding yeast) and its homologs Fft3 (fission yeast) and SMARCAD1 (human; Etl1 in mouse) are non-essential Snf2-like Etl1-subfamily nucleosome remodelers which function in DNA replication, heterochromatin stability, transcription, meiotic hotspot activity, and regulation of DNA repair (Flaus, 2006; Okazaki et al., 2008; Neves-Costa et al., 2009; Rowbotham et al., 2011; Strålfors et al., 2011; Yu et al., 2011; Chen et al., 2012; Costelloe et al., 2012; Eapen et al., 2012; Byeon et al., 2013; Steglich et al., 2015; Densham et al., 2016; Doiguchi et al., 2016; Bantele et al., 2017; Lee et al., 2017; Xiao et al., 2017; Chakraborty et al., 2018; Ding et al., 2018; Jahn et al., 2018; Storey et al., 2018; Terui et al., 2018; Sachs et al., 2019). Notably, during DNA double-strand break (DSB) repair a major function of Fun30 orthologs appears to be in DNA end resection, a process that requires the mobilization and likely eviction of nucleosomes (Chen et al., 2012; Costelloe et al., 2012; Eapen et al., 2012; Bantele et al., 2017). In apparent contrast, during DNA replication of heterochromatin, Fun30 orthologs in fission yeast and human cells seem to rather provide stability of nucleosomes and to prevent loss of heterochromatic histone marks (Rowbotham et al., 2011; Taneja et al., 2017).

With this review, we aim to summarize current data in order to show commonalities and highlight regulatory mechanisms controlling Fun30^SMARCAD1^ remodelers with a special focus on the DNA damage response. The different Fun30^SMARCAD1^ functions appear discrepant at first view, but in this review we will also attempt to point toward commonalities.

## Domain Structure of Fun30 and its Orthologs

Fun30 and its orthologs are ~1,000 amino acids large, single-subunit nucleosome remodelers, which appear to act in homodimeric form (Awad et al., 2010). A bioinformatic analysis showed that Fun30 shares the highest degree of homology with Swr1 and Ino80 of the Snf2 remodeler family (Flaus, 2006). It comprises the catalytic Snf2 nucleosome remodeling domain, but with a Fun30-specific yet uncharacterized insert at the C-terminus (Liu and Jiang, 2017). The N-terminal half of the protein appears to be regulatory and harbors specific regions with the ability to engage in protein-protein interactions (Flaus, 2006; Neves-Costa et al., 2009; Bantele et al., 2017). At the N-terminus, conserved Cyclin-dependent kinase (CDK) phosphorylation sites in yeast Fun30 and human SMARCAD1 (Chen X. et al., 2016; Bantele et al., 2017) are followed by ubiquitin-binding CUE (Coupling of Ubiquitin conjugation to ER degradation) domains, which exist in one or more copies in almost all Fun30 orthologs. In human SMARCAD1, further regulatory ATM phosphorylation sites and phosphorylation-dependent RING1 ubiquitylation sites are targeted after DNA damage and located at the C-terminus (Matsuoka et al., 2007; Densham et al., 2016; Chakraborty et al., 2018). In the following, we will view Fun30^SMARCAD1^ from N to C and summarize the molecular role of the additional regulatory elements.

### CDK Phosphorylation at the N-Terminus of Fun30 and SMARCAD1

Several studies have established Fun30 as CDK substrate *in vitro* and *in vivo* (Ubersax et al., 2003; Chen et al., 2012; Chen X. et al., 2016; Bantele et al., 2017). Specifically, Fun30 is targeted by CDK on S20, S28, and S34 (Chen X. et al., 2016; Bantele et al., 2017). Similarly, SMARCAD1 can be phosphorylated by CDK on T71 (Bantele et al., 2017). Once phosphorylated, S20 and S28 in Fun30 and T71 in SMARCAD1 mediate a direct protein-protein interaction with the N-terminal BRCT repeats of the scaffold protein Dpb11 (in yeast) and TOPBP1 (in human) (Bantele et al., 2017). In yeast, this interaction leads to formation of a ternary complex with the 9-1-1 checkpoint clamp and contributes to targeting Fun30 to sites of DNA damage (Bantele et al., 2017, Figure 1). These data suggest that phosphorylation is a means to localize Fun30, but additionally it is possible that phosphorylation and the associated protein-protein interactions are involved in activating the remodeller toward its substrate.

**Figure 1:**
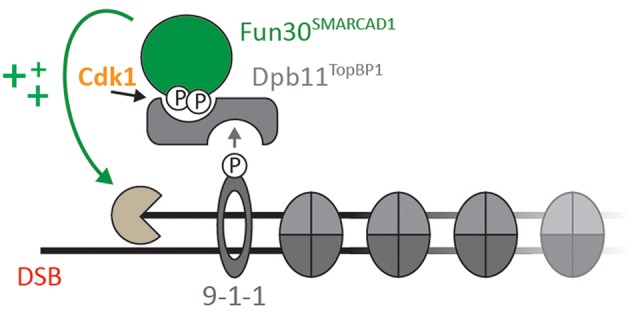
Cell cycle- and DNA damage-activated kinases lead to formation of a ternary complex formed by Fun30^SMARCAD1^, Dpb11^TOPBP1^, and the 9-1-1 complex (adapted from Bantele et al., 2017; Bantele, 2018). Upon CDK-dependent phosphorylation of Fun30 S20/S28 or SMARCAD1 T71, respectively, Fun30 and SMARCAD1 associate with BRCT1+2 of Dpb11 or BRCT0/1/2 of TOPBP1. In yeast, binding to the 9-1-1 complex (in a DNA damage-induced manner) contributes to localization of Fun30-Dpb11 to sites of DNA end resection, where it stimulates long-range resection (Chen et al., 2012; Costelloe et al., 2012; Eapen et al., 2012; Bantele et al., 2017).

### CUE Domains

CUE domains are known for their ability to bind ubiquitin (Donaldson et al., 2003; Kang et al., 2003; Shih et al., 2003), and the N-terminal CUE domain in SMARCAD1 was shown to mediate interactions with the chromatin regulator KAP1 (Rowbotham et al., 2011; Ding et al., 2018; Lim et al., 2019). Furthermore, a CUE domain-dependent interaction of SMARCAD1 with ubiquitylated histone H2A has been identified recently (Densham et al., 2016). Whether binding of ubiquitylated histones is a conserved property of Fun30 orthologs remains to be shown. While a CUE domain has been bioinformatically predicted for Fun30 as well (Neves-Costa et al., 2009), so far no binding partner of the Fun30 CUE domain could be identified. *In vitro* experiments also failed to provide evidence for Fun30 binding to ubiquitylated histones (Awad et al., 2010). Nonetheless, the CUE-dependent protein-protein interactions seem to contribute to context-dependent chromatin localization in the human protein (Densham et al., 2016; Ding et al., 2018). Interestingly, the SMARCAD1-KAP1 interaction has very recently been shown to occur between the SMARCAD1 N-terminal CUE domain and a specific patch in KAP1 that does structurally not resemble ubiquitin (Lim et al., 2019). This finding suggests alternative and still-to-be explored interaction modes of Fun30^SMARCAD1^.

### DNA Damage-Dependent Phosphorylation at the C-Terminus of SMARCAD1

SMARCAD1 is a substrate of the ATM kinase and gets phosphorylated on T906 upon DNA damage (Matsuoka et al., 2007; Densham et al., 2016; Chakraborty et al., 2018). This modification is a prerequisite for the subsequent ubiquitylation on K905 in a RING1-dependent manner (Chakraborty et al., 2018). Both, DNA damage-dependent phosphorylation and ubiquitylation of SMARCAD1 were connected to functions in the DNA damage response, but do not seem to be conserved in the yeast protein. Interestingly, Fun30 was suggested to interact with other proteins of the DNA damage response, such as DNA end resection enzymes Exo1 and Dna2, as well as RPA (Chen et al., 2012). Where the specific interaction sites are located, whether all interactions are direct and in how far they are regulated by post-translational modification remains to be determined.

## Biochemical Activities of Fun30^SMARCAD1^

Nucleosome remodelers use ATP to remodel histone-DNA contacts in order to move or position nucleosomes, evict them or change their composition (Clapier and Cairns, 2009; Hargreaves and Crabtree, 2011). These molecular activities can be studied well *in vitro* and analogous experiments have been performed for Fun30 (Awad et al., 2010; Adkins et al., 2013, 2017).

Fun30 has the general ability to directly bind DNA *in vitro*. Interestingly, both single-stranded (ss) and double-stranded (ds) DNA as well as nucleosome-associated DNA was bound (Awad et al., 2010; Adkins et al., 2017). In line with these findings, presence of ssDNA, dsDNA, or chromatin stimulated the ATPase activity of Fun30 as was observed for other remodelers (Awad et al., 2010; reviewed in Zhou et al., 2016; Clapier et al., 2017). *In vitro*, nucleosomes were seen to be repositioned in the presence of Fun30 and H2A-H2B dimers were found to be liberated from chromatin templates, suggesting that Fun30 has nucleosome sliding and histone dimer exchange activity (Awad et al., 2010; Byeon et al., 2013). *In vivo*, evidence for a dimer exchange activity of Fun30 is currently lacking, but *fun30*Δ cells showed alterations in the nucleosome-free region at the 5′ end of gene bodies, as well as altered occupancy of −1, +2, and +3 nucleosomes (Byeon et al., 2013). Consistently, also fission yeast Fft3 was found to be required for chromatin architecture (Durand-Dubief et al., 2012). Overall, these data are in good agreement with a role of Fun30 and its orthologs in nucleosome sliding and perhaps positioning, but at this point indirect effects on cellular chromatin architecture cannot be ruled out.

In the context of DNA damage, it is unknown whether Fun30 is involved in nucleosome sliding and/or positioning or whether it plays other roles. While a previous study did not find evidence for Fun30 mediating changes in nucleosome positioning in the proximity of a DSB (Costelloe et al., 2012), it might be technically challenging to visualize such changes during dynamic repair.

A possible role in H2A-H2B dimer exchange may manifest in changes in occupancy of the H2A variant H2A.Z or perhaps also of post-translationally modified forms of H2A (such as γH2A). Distribution of H2A.Z was indeed influenced by Fft3 and Fun30, both genome-wide and particularly in centromeric, pericentromeric, and subtelomeric chromatin (Strålfors et al., 2011; Durand-Dubief et al., 2012). Given that H2A.Z is a well-known regulator of DSB repair (van Attikum et al., 2007; Kalocsay et al., 2009; Xu et al., 2012; Adkins et al., 2013; Lademann et al., 2017), it is tempting to hypothesize that Fun30 regulates H2A.Z at DSBs as well. So far, it remains to be determined whether the changes in H2A.Z distribution induced by Fun30 occur at DSBs and might potentially contribute to resection regulation.

Notably, Fun30 has particular binding preferences when it comes to nucleosome structure and modification, for example it seems to be repelled by S129-phosphorylated H2A (γH2A, induced by DNA damage) (Eapen et al., 2012; Adkins et al., 2017). One could therefore speculate that Fun30 might antagonize γH2A via an H2A/H2B dimer exchange activity. Experimental data however argue against such a model, as no changes in γH2A phosphorylation after DNA damage could be observed in mutants lacking Fun30 (Eapen et al., 2012).

Lastly, during maintenance of heterochromatin/ transcriptionally silent chromatin, Fun30/Fft3/SMARCAD1 appear to function as stabilizers of chromatin marks (Durand-Dubief et al., 2012; Byeon et al., 2013; Taneja et al., 2017), but whether this can be explained by sliding/dimer exchange activities or whether this function involves an additional activity is not known.

Overall impressive progress has been made toward understanding the catalytic activities of Fun30^SMARCAD1^, but nonetheless we currently do not understand the specific nature of the substrate toward which the remodeling activity is directed to, nucleosomes or modified nucleosomes are a possibility, but the function in DSB repair (see below) suggests that it might also be nucleosomes in complex with an additional protein(s) or maybe even a nucleosome-bound protein.

## Biological Functions of Fun30^SMARCAD1^

At first glance, the biological functions of Fun30 and its orthologs appear at least as diverse as its biochemical activities (sliding, positioning, dimer exchange). In the following, we therefore aim to not only summarize the known functions, but also to highlight commonalities, since a common model describing Fun30 function would help discriminate direct from indirect consequences of a loss of Fun30 function and facilitate future research.

### Gene Expression Control

Orthologs of Fun30 promote gene expression. Fission yeast Fft3 facilitates the progression of RNA Polymerase II through actively transcribed genes by mediating nucleosome dissociation (Lee et al., 2017). Also, SMARCAD1 was found to act as transcriptional activator and enhances CBP-mediated histone acetylation (Doiguchi et al., 2016). The overall importance of the contribution of Fun30 orthologs to transcription regulation remains however to be elucidated. At least in budding yeast, absence of Fun30 caused only minor changes in the expression of few proteins (Chen et al., 2012), possibly reflecting redundancy with other nucleosome remodelers (Barbaric et al., 2007; Smolle et al., 2012).

### Maintenance of Silent Chromatin

All Fun30 orthologs were shown to localize to heterochromatic or repressed genomic loci and contribute to their establishment and preservation. Fission yeast Fft3 and budding yeast Fun30 localize to insulator elements and are involved in silencing at subtelomeres, centromeres, rDNA repeats, and mating type loci (Neves-Costa et al., 2009; Strålfors et al., 2011; Durand-Dubief et al., 2012; Steglich et al., 2015; Taneja et al., 2017; Jahn et al., 2018). In absence of Fft3, the composition and nuclear localization of heterochromatin is altered and accumulates euchromatic histone modifications such as H4K12Ac and H3K9Ac, as well as histone variants like H2A.Z (Strålfors et al., 2011; Steglich et al., 2015). Fun30 contributes to transcriptional repression of genes and across centromeres in order to ensure unhampered chromosome segregation (Strålfors et al., 2011; Durand-Dubief et al., 2012; Byeon et al., 2013). *In vivo*, Fft3, Fun30, and SMARCAD1 thus seem to ensure maintenance and inheritance of boundaries between chromatin states by stabilizing nucleosomes and preserving heterochromatic histone marks (Durand-Dubief et al., 2012; Byeon et al., 2013; Taneja et al., 2017; Xiao et al., 2017; Ding et al., 2018; Sachs et al., 2019).

Notably, Fun30 and SMARCAD1 are not only involved in maintenance of heterochromatin or silent chromatin, but are also involved in generating repressed chromatin *de novo*, where an interaction with HDAC1/2 mediating H3/H4 deacetylation might be involved (Okazaki et al., 2008; Rowbotham et al., 2011; Yu et al., 2011).

Inheritance of nucleosomes is crucial for heterochromatin maintenance and therefore is tightly linked to DNA replication (Saredi et al., 2016; Yadav and Whitehouse, 2016; Yang et al., 2016; reviewed in Serra-Cardona and Zhang, 2018). In line with this, it was not only shown that SMARCAD1 is required for heterochromatin maintenance in proliferating cells, but SMARCAD1 was also shown to bind to the replication factor PCNA (Rowbotham et al., 2011) suggesting a possible mechanism for how it could be targeted to sites of DNA replication. Also, the CUE domains of SMARCAD1 are specifically required and could play a role in targeting (Rowbotham et al., 2011; Ding et al., 2018). The first CUE domain of SMARCAD1 binds to KAP1 (Ding et al., 2018), but a universal function of the CUE domains as well as the link between CUE-dependent interactors and PCNA has not been established. A putative role of Fun30^SMARCAD1^ in chromatin inheritance during DNA replication is also interesting, since DNA replication involves formation of ssDNA and nucleosome eviction and therefore features mechanistic similarities to DNA resection, another process where Fun30 is crucially involved in (see below).

### DNA Damage Response and DSB Repair

First connections of Fun30 to the DNA damage response were made by several genetic screens in budding yeast—a screen identifying factors involved in chromosome stability and segregation (Ouspenski et al., 1999), several genetic interaction screens with DNA repair mutants (Krogan et al., 2003, 2006; Collins et al., 2007; Beltrao et al., 2009; Costanzo et al., 2010), a screen for mutants affecting gene targeting (Chen et al., 2012), and a screen for mutants affecting break-induced replication (Costelloe et al., 2012). Fun30 and SMARCAD1 were furthermore connected to the DNA mismatch repair pathway (MMR; Chen Z. et al., 2016; Goellner et al., 2018; Terui et al., 2018) and shown to be required for the resistance to irradiation and camptothecin (CPT) (Neves-Costa et al., 2009; Costelloe et al., 2012; Chakraborty et al., 2018). In 2012, a series of pioneering publications established a key role of Fun30 and SMARCAD1 during the repair of DNA DSBs by homologous recombination (Chen et al., 2012; Costelloe et al., 2012; Eapen et al., 2012). Together with more recent work (Chen X. et al., 2016; Densham et al., 2016; Bantele et al., 2017) these publications convincingly demonstrate a molecular function in promoting DNA end resection, the nucleolytic digestion of dsDNA at DSBs that leads to the formation of 3′ssDNA overhangs.

#### Enhancement of DSB Resection

DNA DSBs can be repaired by non-homologous end-joining (NHEJ) or homologous recombination (HR) pathways (Symington and Gautier, 2011). The choice between these two repair regimes depends strongly on the cell cycle state and is determined at the step of DNA end resection, where DSB ends are nucleolytically digested so that 3′ overhangs are formed. These overhangs constitute crucial intermediates of repair by homologous recombination and moreover have a central signaling function at DSBs.

It is reasonable to assume that nucleosomes constitute a barrier to DNA end resection into undamaged chromatin, and indeed chromatinized DNA is resected less efficiently with increasing nucleosome density *in vitro* (Adkins et al., 2013). Notably, two nuclease complexes are mainly responsible for spreading of resection (long range resection) (Zhu et al., 2008; Mimitou and Symington, 2009). These nucleases—Exo1 alone and Dna2 in conjunction with the Sgs1-Top3-Rmi1 complex—bypass nucleosomes with distinct mechanisms, suggesting that they might require different forms of nucleosome remodeling (Adkins et al., 2013). Furthermore, different chromatin states such as heterochromatin might require additional means to promote resection (Baldeyron et al., 2011; Eapen et al., 2012; Soria and Almouzni, 2013; Batté et al., 2017).

Notably, while Fun30 and SMARCAD1 are required for efficient long-range resection through chromatin *in vivo* (Chen et al., 2012; Costelloe et al., 2012; Eapen et al., 2012; Bantele et al., 2017), initial studies could not demonstrate an effect of Fun30 on resection through chromatinized DNA *in vitro*, at least in case of Exo1 (Adkins et al., 2013). Most likely, the *in vitro* system therefore fails to recapitulate the *in vivo* situation. This allows to speculate that the specific substrate of Fun30 remodeling during DNA end resection might have been missing from the *in vitro* reaction.

In this regard, it is interesting to note that genetics have revealed a major function of Fun30 and SMARCAD1 in counteracting a resection inhibitor—Rad9 in yeast, 53BP1 in humans (Chen et al., 2012; Densham et al., 2016; Bantele et al., 2017). The role of Rad9 and 53BP1 as inhibitors of DNA end resection is clearly established. However, it is not clear whether the specific mechanism of resection inhibition is conserved through evolution. Notably, both Rad9 and 53BP1, as well as fission yeast Crb2 are nucleosome binders and appear to recognize several (modified) histones, suggesting multivalency (Huyen et al., 2004; Nakamura et al., 2004; Sanders et al., 2004; Wysocki et al., 2005; Botuyan et al., 2006; Toh et al., 2006; Grenon et al., 2007; Hammet et al., 2007; Fradet-Turcotte et al., 2013; Wilson et al., 2016). Again, the specific nature of binding sites does not appear to be conserved, but the multivalent interaction with nucleosomes is shared by Rad9 orthologs. In the absence of Rad9 or 53BP1, the remodeling activity of Fun30 or SMARCAD1 seems to be at least partly dispensable and phenotypes such as CPT sensitivity are suppressed (Chen et al., 2012; Densham et al., 2016; Bantele et al., 2017). Collectively, these data establish Rad9^53BP1^-bound nucleosomes as excellent candidate substrate for Fun30 activity. Consistently, Rad9^53BP1^ was shown to accumulate around DSBs when Fun30^SMARCAD1^ was absent (Chen et al., 2012; Costelloe et al., 2012; Densham et al., 2016). In both yeast and human cells, the ATPase activity of the remodeler is required to facilitate resection, implying active remodeling as part of the resection-promoting mechanism (Bantele et al., 2017; Chakraborty et al., 2018). One can therefore conclude that the opposition to Rad9^53BP1^ is a central task of Fun30^SMARCAD1^-dependent remodeling.

**Figure 2:**
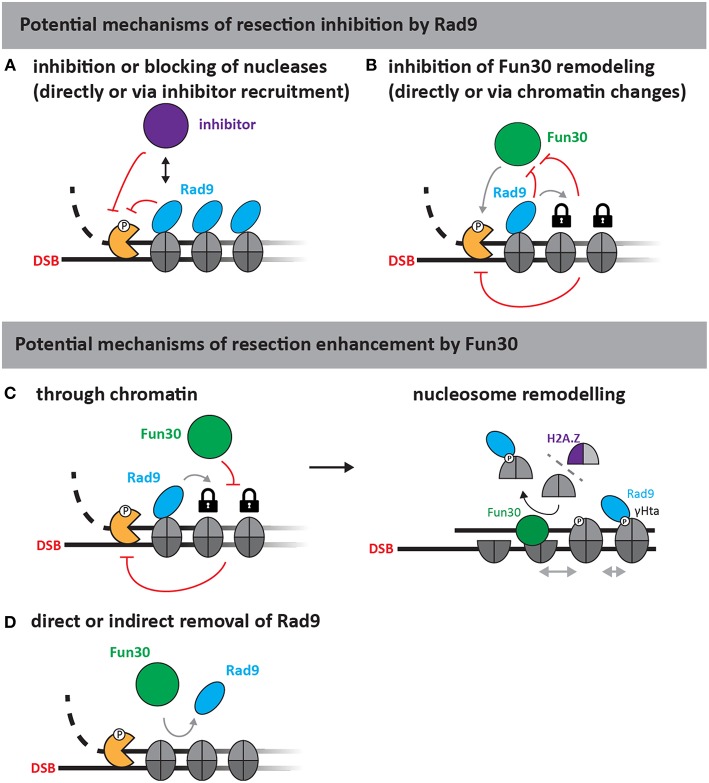
Putative mechanisms of resection regulation by Fun30 and Rad9 (adapted from Bantele, 2018). As Rad9 is a chromatin-binding protein without apparent catalytic activity, at least two mechanisms of resection inhibition can be envisioned (upper part). First, Rad9 could directly block or slow down the progression of nucleases either by inhibiting the nucleases **(A)** or by stabilizing chromatin in a configuration that is non-permissive to resection **(B)** for example by inhibiting Fun30, if the latter was required to help overcome resection-inhibition by nucleosomes. Fun30 could also promote resection by several different mechanisms (lower part). As a nucleosome remodeler, Fun30 could either act through chromatin **(C)**, or by removing Rad9 from chromatin **(D)**. The action through chromatin could involve its putative remodeling activities and potentially H2A/H2B dimer exchange, which might affect γH2A and H2A.Z dynamics or repositioning of nucleosomes by nucleosome sliding (**C**, right side).

The molecular nature of the Rad9^53BP1^-Fun30^SMARCAD1^ antagonism is currently elusive. Figure 2 highlights plausible models for the yeast proteins, where Rad9 could act as specific inhibitor of resection, directly or indirectly inhibiting the resection nucleases via chromatin, for example by inhibiting Fun30 (Figures 2A,B). Conversely, Fun30 might overcome resection-inhibition by Rad9 and in this instance either directly remove Rad9 from chromatin, block its association in an indirect manner or counteract its downstream effects (Figures 2C,D). Since Rad9 is a nucleosome-binder, an indirect effect on Rad9 association could depend on the histone marks recognized by Rad9. For example, removal of the γH2A phosphorylation mark would lead to a defect in Rad9 chromatin association (Javaheri et al., 2006; Hammet et al., 2007; Eapen et al., 2012; Clerici et al., 2014). Such a model would be consistent with the described H2A-H2B dimer exchange ability of Fun30 (Awad et al., 2010), but would be inconsistent with experimental data, where cells lacking the γH2A modification still partially require Fun30 for efficient resection (Eapen et al., 2012). We anticipate that biochemical reconstitution will identify the remodeling substrate of Fun30^SMARCAD1^ and allow to reveal the mechanism by which Rad9^53BP1^ and Fun30^SMARCAD1^ antagonize each other.

It is also interesting to note that several of the binding partners of Fun30 are shared by its antagonist Rad9. These include histones, but also the BRCT repeat protein Dpb11 (Javaheri et al., 2006; Hammet et al., 2007; Pfander and Diffley, 2011; Bantele et al., 2017). Notably, in case of Dpb11, Fun30 and Rad9 even share the binding site, suggesting direct competition (Bantele et al., 2017). However, while competition might contribute to the antagonistic relationship, it is certainly not the exclusive source of this antagonism, as resection depends on the catalytic activity of Fun30 even in a context of a Fun30-Dpb11 fusion protein (Bantele et al., 2017).

Also in human cells, SMARCAD1 antagonizes 53BP1, as depletion of SMARCAD1 leads to a stabilization of 53BP1 at DSBs (Densham et al., 2016). Compared to the budding yeast system, the situation in human cells appears to be more complex. First, human cells have a second, well-established pro-resection factor and 53BP1 antagonist—BRCA1 (Cao et al., 2009; Bothmer et al., 2010; Bouwman et al., 2010; Bunting et al., 2010, 2012; Escribano-Díaz et al., 2013). BRCA1 forms a ubiquitin ligase complex together with BARD1 and BRCA1-BARD1 were shown to mediate ubiquitylation of histone H2A (Kalb et al., 2014; Densham et al., 2016). Notably, this might be a point where the two pro-resection pathways converge, since ubiquitylated H2A appears to stabilize SMARCAD1 at the DSB site, likely via direct CUE domain-dependent binding of SMARCAD1 to ubiquitylated H2A (Densham et al., 2016). A second layer of complexity comes in the form of 53BP1 effectors, such as RIF1, REV3, and the Shieldin complex (Xu et al., 2015; Dev et al., 2018; Findlay et al., 2018; Gupta et al., 2018; Mirman et al., 2018; Noordermeer et al., 2018). These effectors may inhibit resection by changing PTMs on damaged chromatin (RIF1 is a PP1 phosphatase-associated factor) or even promoting fill-in DNA synthesis (Hiraga et al., 2014; Mirman et al., 2018; Bhowmick et al., 2019; Garzón et al., 2019). Similar mechanisms have not yet been described in yeast and might represent metazoan-specific additions to an evolutionary conserved chromatin-dependent control of DNA end resection.

#### Cell Cycle Control and DSB Repair Pathway Choice

DSB repair by homologous recombination is coupled to the presence of a sister chromatid and therefore DSB repair pathway choice is cell cycle-regulated. This cell cycle-regulation impinges on the control of resection by Fun30, as Fun30 is phosphorylated by CDK (Chen X. et al., 2016; Bantele et al., 2017). Mechanistically, CDK-phosphorylation generates a binding site for the scaffold protein Dpb11, which in turn binds to the 9-1-1 checkpoint clamp thus leading to a ternary complex between Fun30, Dpb11, and 9-1-1 (Figure 1; Bantele et al., 2017). Formation of this complex mediates targeting to and likely activation of Fun30 at sites of DNA end resection (Bantele et al., 2017). A similar mechanism is likely occurring also in mammalian cells as SMARCAD1 can be phosphorylated by CDK as well, leading to interaction with the Dpb11 ortholog TOPBP1 (Bantele et al., 2017).

Budding yeast cells arrested in M phase show DNA end resection of DSBs that strongly depends on Fun30 and the Fun30 targeting complex (Bantele et al., 2017). Nonetheless, additional factors are clearly involved in the cell cycle control of DNA end resection. Overall, these findings raise the question of which specific DNA end resection pathway and pathway decision Fun30 is actually involved in. In this regard it has been shown that repair by homologous recombination requires resection of only a few 100 base pairs (Jinks-Robertson et al., 1993; Ira and Haber, 2002; Zhu et al., 2008), while for alternative recombination pathways and repair by single-strand annealing (SSA) in particular longer stretches of resected DNA are required (Zhu et al., 2008). One can therefore reason that the switch between NHEJ and HR is already done once resection initiates and that activation of long-range resection by Fun30 would rather further shift repair to an SSA-type mechanism. Indeed, a mild decrease of DNA end resection efficiency for example in an *exo1*Δ or *fun30*Δ mutant strain seems beneficial for HR efficiency (Lee et al., 2016), while it impedes SSA repair (Chen et al., 2012; Eapen et al., 2012; Bantele et al., 2017). Already now, one can however conclude that the Fun30-Rad9 switch and its effect on the DSB surrounding chromatin adds a further layer to DSB repair pathway choice and its changing nature during the cell cycle. It will be exciting to explore, in how far genetic tools such as a fusion of Fun30 to the 9-1-1 complex (Bantele et al., 2017) can be used to bypass these controls and whether they can be utilized for HR-dependent genome editing reactions.

### Role During Chromatin Disruption and Regulation by DNA Clamps

Past research has given us very valuable insights into the individual functions of Fun30^SMARCAD1^, but is there commonality or can we link them to a specific enzymatic activity? The most obvious commonality at least between the function during DNA end resection and the function in the maintenance of silent chromatin regions during DNA replication is that both DNA end resection and DNA replication involve the formation of ssDNA and eviction of nucleosomes. While neither Fun30 nor any of its orthologs have been directly tested for an “evictase” function, the presence of Fun30 did not overcome the barrier function of nucleosomes toward the Exo1 exonuclease, arguing that Fun30 is not an “evictase” (Adkins et al., 2013). Moreover, the function of Fun30^SMARCAD1^ at DSBs and replication forks seems generally discordant, since during DNA end resection and most likely also transcription Fun30^SMARCAD1^ seems to open-up chromatin, while during replication it rather seems to be involved in stabilizing nucleosomes (Rowbotham et al., 2011; Chen et al., 2012; Costelloe et al., 2012; Eapen et al., 2012; Lee et al., 2018).

Nonetheless, there is more commonality—in particular seen in the regulation of Fun30^SMARCAD1^ by DNA clamps. During resection, Fun30 acts in complex with the 9-1-1 clamp (connected by the Dpb11 bridge) and this complex is likely conserved in humans as well (Takeishi et al., 2010; Ohashi et al., 2014; Bantele et al., 2017). Strikingly, SMARCAD1 was also found to bind to PCNA (Rowbotham et al., 2011), a processivity factor during DNA replication and key platform for protein recruitment at replication forks (Moldovan et al., 2007). While this similarity is striking, the connection to PCNA and 9-1-1 can in fact rather offer an explanation for the discrepant roles of Fun30^SMARCAD1^ during replication and DSB resection. PCNA and 9-1-1 were shown to be loaded onto double-stranded DNA in *in vitro* experiments and our unpublished data suggest that the same is true *in vivo* (Gomes and Burgers, 2001; Gomes et al., 2001; Majka et al., 2006; reviewed in Majka and Burgers, 2004; Peritore and Pfander, unpublished). Interestingly, the DNA clamps are located at very different positions, if one compares a replication fork to sites of DSB resection. At sites of resection, dsDNA is present upstream of the ss-dsDNA junction. The 9-1-1 complex and associated factors are therefore loaded “in front” of the resecting nucleases (see Figure 1). By associating with the 9-1-1 complex, Fun30^SMARCAD1^ is therefore in an ideal position to remove potential obstacles ahead of the resecting nucleases. PCNA in turn is loaded at the primer-template junction and as such will travel behind the replicative polymerase and helicase (Moldovan et al., 2007). As such, binding to PCNA will allow SMARCAD1 not only to associate with the replisome, but exactly to the place where chromatin is restored (Rowbotham et al., 2011). Thus, while similar mechanisms are used to control Fun30^SMARCAD1^ in different processes, the combination of localization and activity leads to different or discordant outcomes.

## Concluding Remarks and Future Research

Fun30 has a key role in promoting DNA end resection and is differentially regulated at different cell cycle stages. Such regulation appears to be of high importance for the maintenance of genomic integrity, and accordingly deregulation of the human ortholog of Fun30, SMARCAD1, was found to play a crucial role during the progression of triple-negative breast cancer, which is specifically characterized by an HR-defect (Kubaisy et al., 2016; Arafat et al., 2018).

It will therefore be highly exciting for future research to further unravel the different functions and molecular mechanisms that Fun30^SMARCAD1^ employ to promote genome integrity. Notably, Fun30 is involved in several aspects of DNA metabolism. The major task here will be to elucidate commonalities and differences between the underlying mechanisms in order to achieve an overarching understanding of Fun30 remodeling activity.

## Author Contributions

BP and SB wrote the review.

### Conflict of Interest Statement

The authors declare that the research was conducted in the absence of any commercial or financial relationships that could be construed as a potential conflict of interest.
